# Factors that influence snacking behaviors among urban Indian adolescents – a qualitative inquiry

**DOI:** 10.3389/fnut.2025.1637799

**Published:** 2025-09-15

**Authors:** Neha Rathi, Anthony Worsley, Meg Bruening

**Affiliations:** ^1^Bagchi School of Public Health, Ahmedabad University, Ahmedabad, India; ^2^School of Nutrition & Dietetics, Symbiosis Skills and Professional University, Pimpri-Chinchwad, India; ^3^Department of Home Science, Mahila Mahavidyalaya, Banaras Hindu University, Varanasi, India; ^4^School of Exercise and Nutrition Sciences, Deakin University, Geelong, VIC, Australia; ^5^Department of Nutritional Sciences, College of Health and Human Development, The Pennsylvania State University, University Park, PA, United States

**Keywords:** qualitative research, India, adolescents, snacking, interviews

## Abstract

**Background and objectives:**

Urban Indian adolescents often practice unhealthy dietary behaviors such as meal skipping and snacking on high energy foods with low nutrient value. To promote healthy snacking behaviors among adolescents it is essential to explore the factors that may influence the consumption of healthy and unhealthy snacks among Indian adolescents.

**Materials and methods:**

Informed by the social constructivism framework, an exploratory-descriptive qualitative research approach was employed to obtain a richer understanding of the influences on urban Indian adolescents’ snacking behaviors. Using purposive sampling, adolescents aged 10–19 years were recruited from two government and two private schools in Varanasi city, Uttar Pradesh, India. Face-to-face interviews were conducted in Hindi/English, per the preference of the participants. The conversations were digitally recorded, transcribed verbatim, and translated to English (where necessary) for analysis. Themes were extracted using inductive coding.

**Results:**

A total of 62 adolescents (50% female; 76% private school pupils) with mean age 15.3 (SD: 1.86) years completed the interviews between November 2024 and February 2025. Ten themes emerged including: (i) Perceptions of a snack; (ii) liking for unhealthy snacks; (iii) consequences of snacking; (iv) snacking timing; (v) cost of snacks; (vi) parental rules around snacking; (vii) influence of peers; (viii) school food environment; (ix) neighborhood food environment; (x) food and beverage marketing.

**Conclusion:**

These findings show that multiple factors are likely to influence snacking behaviors in Indian adolescents, aligning with literature on adolescent snack consumption in international settings. Behavioral interventions should be designed to create enabling environments to encourage healthy snack consumption in adolescents by removing barriers at the individual, household, and societal levels.

## Introduction

Adolescent obesity is a global public health crisis with increasing prevalence in low-income and middle-income economies like India (1, 2). During the last decade, India has witnessed a substantial rise in adolescent obesity (3, 4) particularly in urban settings (5), with a varying prevalence ranging from 19.6 to 26.6% (3, 6). Likewise, 22.9% males and 24% females (age: 15–49 years) were diagnosed either as overweight or obese in a recent nationwide survey (7). This mounting prevalence of adolescent obesity is likely to impose an increased disease burden in adulthood as it is associated with psychosocial and cardiovascular morbidity as well as premature mortality (2, 8–10), thus placing a great load on India’s healthcare system and economy (1). Therefore, behavior change interventions aimed at reducing adolescent obesity through improved eating behaviors and its associated comorbidities are of national importance (1, 2).

One of the possible behavior change strategies to tackle this emerging health crisis could be the development and maintenance of healthy dietary behaviors. However, both local and international evidence reveals that adolescents practice unhealthy dietary behaviors such as meal skipping and snacking on high energy foods with low nutrient value (11–15). Overconsumption of energy-dense, nutrient-poor (ENDP) snacks is widely cited as one of the potential drivers of the obesity epidemic (11, 16–18). Yet, little research has been conducted to understand snacking behaviors among Indian adolescents.

In contrast, snacking on healthy foods and drinks like legume-based snacks, fruits, and low-fat dairy is known to promote appetite control, thereby reducing the risk of adiposity (19, 20). In addition, Leidy and colleagues noted that consumption of nutrient dense snacks in the afternoon was associated with appetite control, improved satiety, and overall diet quality in adolescent boys (21). In the same vein, mid-morning and mid-afternoon snacking on healthy foods is known to alleviate the potential digestive and metabolic overload caused by three large meals (i.e., breakfast, lunch, and dinner) (22) as well as assist consumers to meet their daily recommendations for food groups (i.e., fruits, dairy) which are sparingly consumed on a daily basis (23). This healthy snacking is further reflected in the Scientific Statement released by the American Heart Association which recommends consumption of smaller portions of food spread across four to six eating occasions every 2–3 h over three main daily meals (24). Considering these benefits associated with healthy snacking, public health specialists strongly endorse the development and implementation of snacking interventions to encourage adolescents to consume healthy snacks and drinks between meals (23, 25, 26).

As part of the development of interventions to encourage healthy snacking in adolescents, it is important to understand the contextual factors that influence adolescents’ snacking behaviors. Nutrition researchers have identified a range of factors including individual (intrapersonal), social environmental (interpersonal), physical environmental (community settings), and societal (macrosystem) factors influencing adolescents’ snacking behaviors (25, 27–34). Some common intrapersonal predictors of snacking include taste preferences, gender, socio-economic status, body image, health attitude and behavioral factors like self-efficacy and autonomy (25, 27, 28, 35, 36).

At the interpersonal level, both family and peer groups play a significant role in determining snacking behavior of adolescents through family food rules and implicit peer pressure, respectively (28–31, 37, 38). Community settings also play a crucial role in influencing snacking, with the school food environment being of prime importance because of the provision of snacks in school canteens (39–42). At the macrosystem level, mass media and associated food marketing are recognized as the most prominent determinants of adolescent snacking behavior (36, 43–45).

Unfortunately, many of these emerging findings about the influences over snacking behaviors in adolescence are predominantly limited to developed economies like the United States (28), the United Kingdom (46), Ireland (25), and Australia (44). As such, they may or may not be applicable to developing economies like India which have different socio-cultural structures and dietary landscapes. For example, the consumption of meat and meat products is more popular in the Western world (e.g., USA, Germany) when compared to India because of the dominance of religion triggered vegetarianism in the Indian dietary landscape (47–49). Moreover, in addressing the emerging obesity burden in India, there is a need for nutrition interventions that account for unique Indian socio-cultural influences on snacking behaviors. Therefore, the present study was designed to gain novel insights into factors that may impact snacking behaviors of Indian adolescents through in-depth interviews. The use of in-depth qualitative research methods was warranted in the present context as they allow for the elucidation of diverse factors including culture-specific influences that impact adolescent snacking behavior which may not be adequately captured through quantitative research techniques. The emerging findings will guide the development of effective and sustainable nutrition interventions specially harnessing the facilitators and overcoming the barriers to healthy snacking and thereby potentially reducing overweight and obesity in Indian adolescents.

## Materials and methods

### Research design

Informed by the sociological theory of social constructivism, an exploratory-descriptive qualitative research approach (50) was employed in the present inquiry to obtain a richer understanding of the influences on adolescents’ snacking behaviors. The social constructivist paradigm provided the investigators with the opportunity to anticipate and appreciate the complexity of respondents’ views about the subject matter which is derived from their interactions with others (51). Using this social constructivist perspective, the study investigators examined the adolescents’ responses to draw novel insights on the factors influencing their snacking behaviors. Ethical approval (EC/2817) for this inquiry was granted by the Ethics Committee of Banaras Hindu University. The Consolidated Criteria for Reporting Qualitative Research (COREQ) – a 32-item checklist (52) was administered to report this inquiry (Supplementary File 1).

### Research team

The research team comprised two female researchers (NR, MB) and a male researcher (AW). The senior author NR is a mid-career scientist from India with expertise in community nutrition and qualitative research methods. She carried out all the interviews and did not have any prior association with the interviewees. Based in the United States, MB was a senior professor with a rich experience in adolescent nutrition. AW is a retired professor of food psychology from Australia. Along with NR, AW and MB were involved in data coding and report writing. This collaboration limited the probability of any individual, gender, or professional biases incurred during thematic coding.

### Sample and recruitment

The adolescents were recruited using purposive sampling from two public (government, no fee payment; mostly attended by pupils belonging to lower socio-economic class) and two private (independent; fee-paying; mostly attended by pupils belonging to higher socio-economic class) schools in Varanasi. The city of Varanasi is located in the south-eastern part of the most populous state in India, Uttar Pradesh. According to census data of 2011, Varanasi was home to 3.6 million individuals (48% females) with an overall literacy rate of 75.60% (53). The main investigator first met the school principals in person to seek their permission to conduct the present inquiry with their pupils during school hours. She introduced this study to the pupils during morning assembly. Pupils were asked to share their interest with their respective class teachers. Both parental consent and adolescent assent forms along with a study information sheet were shared with the interested students. Both the forms and the information sheet were printed in Hindi as well as English. Students were asked to return the completed forms to the researcher through their class teachers and subsequently, the researcher coordinated with the class teacher about the date and time of interviews.

Efforts were made to recruit relatively equal numbers of adolescent boys and girls since snacking behaviors may be influenced by gender (29, 54, 55) as adolescent boys tend to consume more unhealthy snacks and beverages compared to adolescent girls (13, 29, 49). Both government and non-government school children were invited to participate in this inquiry because of the potential influence of the food environment on snacking behavior (28, 42). The food environment in government schools differs from that in non-government schools in India (i.e., free hot lunches are available in public schools till eighth grade while most private schools host school canteens where students can purchase their meals and snacks) (56).

### Data collection

Based on existing literature (28), the semi-structured interview guide comprising 11 open-ended questions (Supplementary File 2) and relevant probes (e.g., What about when you’re at a grocery store?) were developed both in English as well as in Hindi and was reviewed for face validity by a Home Economics school educator as well as a Public Health Nutritionist. The guide was pilot-tested with four adolescents (2 boys and 2 girls) to verify readability and comprehension before commencement of data collection. In addition to the open-ended questions, basic socio-demographic information like age, gender, type of school and grade was also collected from the interviewees.

In the presence of a note keeper, semi-structured, face-to-face interviews were conducted by the lead author (NR) in Hindi as well as English as per the preferences of the interviewees (NR is fluent in both the languages). All the interviews were conducted in a closed room in the school building where no classmate or educator known to the participant was present. Field notes were taken by the note keeper. Data saturation (57) was attained at the 62^*nd*^ interview and subsequently data collection ceased. Upon completion of the interviews, the interviewees received fruits (i.e., guava, banana, and orange) and plain yoghurt. The interviews were digitally recorded, and their duration ranged from 30 to 40 min (Mean: 35.75; SD: 4.34).

### Data analysis

The interview recordings were transcribed verbatim and translated to English (where necessary) by the note keeper (the note keeper was fluent in both English and Hindi) for the purpose of data analysis. The interviewer further checked the transcripts for accuracy against their corresponding digital recordings and subsequently, they were imported to NVivo software program (Version 15, Lumivero 2024) to organize and code the data.

The transcripts were subjected to inductive thematic analysis in a six-phase process (58) as outlined by Braun and Clarke. In the first phase, the transcripts were read multiple times by the lead researcher (NR) for data familiarization, and this was followed by production of initial codes (i.e., interesting features of the data) as well as arranging them in a systematic manner. During the third phase, the initial codes were sorted into possible themes and all the pertinent coded data excerpts were categorized within the identified themes. In the fourth phase, the identified themes were reviewed and refined at two levels (i.e., verifying whether the identified themes were applicable to the coded excerpts in level 1 and a similar verification was adopted in level 2 to confirm the validity of the themes in relation to the entire date set) to form a thematic map. In the fifth phase clear-cut definitions and names for each theme were generated. Lastly, a comprehensive coherent, logical report (manuscript) of the interviewee accounts was created in which the final themes were linked to the reports of the interviewees (58).

To improve reliability and minimize any potential bias arising from personal interpretation of the transcribed data, the transcripts were coded by two coders (NR, MB) (59). Any disagreement between the two coders was resolved through consultation with the third author (AW). Furthermore, respondent validation was performed to ensure fidelity (60). All the adolescents were requested to assess their transcripts but none of them showed any interest in reviewing them.

## Results

### Socio-demographic characteristics of the sample

The final sample comprised 62 participants (50% females) with a mean age of 15.3 years (SD Y: 1.86). Three-fourths of the sample (75.8%) attended private school, and a similar proportion (75.8%) were studying in high school (i.e., between 9^*th*^ and 12^*th*^ grade).

### Themes

Ten major themes were identified through data analysis of the 62 interviews associated with adolescents’ perceptions of snacking behaviors and its determinants: (i) Perceptions of a snack; (ii) liking for unhealthy snacks; (iii) consequences of snacking; (iv) snacking timing; (v) cost of snacks; (vi) parental rules around snacking; (vii) influence of peers; (viii) school food environment; (ix) neighborhood food environment; (x) food and beverage marketing (Table 1).

**TABLE 1:** Themes associated with factors influencing snacking behaviors in adolescents.

Themes	Quotes
Theme 1: Perceptions of a snack	“*Snacks are junk food and fast food we buy from shops.*” (P1, F, 14 years) “*I guess snacking is when we eat between meals and also when we feel hungry.*” (P29, M, 19 years) “*Snacks means something light weight which I eat to satisfy myself in between meals with tea. Snacks are not just eaten for hunger. It’s for satisfaction or for uplifting the mood. Kurkure (A packaged snack containing deep fried corn meal)*” (P21, F, 15 years) “*When there’s anxiety, like when I’m stressed about exams, I start munching on snacks. It makes me feel a bit lighter.*” (P34, F, 17 years)
Theme 2: Liking for unhealthy snacks	“*Actually, very healthy things aren’t tasty.*” (P1, F, 14 years) “*I eat fruits, but I don’t eat that often. I don’t like its taste.*” (P50, F, 15 years) “*Mostly I prefer crunchy and spicy snacks, and I like eating biscuits with chocolate flavor.*” (P24, M, 15 years) “*My favorite is Oreo biscuit. I eat Oreo biscuits to enjoy its taste and flavor.*” (P59, M, 18 years)
Theme 3: Consequences of snacking	“*Snacking can result in obesity, acidity [SIC: heartburn].*” (P2, M, 14 years) “…*Snacks are oily and therefore it is not good for our health.*” (P18, F, 16 years) “*Homemade snacks do not affect our health, but snacks consumed outside can cause fever and stomachache*” (P4, M, 14 years)
Theme 4: Snacking episodes	“*When I’m home, I generally snack more because most of the time I watch movies and while watching movies I love to munch.*” (P52, M, 15 years) “*When I come back from tuition, I usually eat snacks with my friends.*” (P23, F, 15 years) “*I usually snack once in the evening*…*.*” (P5, F, 16 years)
Theme 5: Cost of snacks	“*Kurkure is easily available also, it’s tasty and like it’s under the budget too.*” (P32, F, 19 years) “*I like to eat Kurkure, Lays, cold drinks, and wafers because they are tasty, and you can get it for very little price.*” (P44, M, 15 years) “*I love to eat momos because they are cheap!*” (P43, F, 17 years)
Theme 6: Parental rules around snacking	“…*There are restrictions on certain things. For example, I am not allowed to eat too much Maggi because it has been reported to contain lead and other harmful substances.*” (P38, F, 17 years) “*My father is very particular about my schedule like I just told you from Monday to Friday I must eat healthy food only, on the weekends only I am allowed to snack on junk food. They make sure I eat almonds daily.*” (P37, M, 12 years)
Theme 7: Influence of peers	“*My friends told me to try Campa Cola* (A brand name for carbonated beverage)*.*” (P22, M, 11 years) “*I don’t eat alone. I eat with my friends. All of us like eating Maggi, Namkeen, chips, momos*…*.*” (P1, F, 14 years)
Theme 8: School food environment	“*There is no canteen in my school. They (School authorities) ask us to eat from outside; there is no such canteen in the school where fast food or junk food is served.*” (P20, M, 17 years) “*In canteen, there is no healthy option available. Only snacks are available like biscuits, chocolates, and cold drinks. Earlier, in mid-day meals, we used to get fruits, but now we don’t.*” (P23, F, 15 years) “*There is a canteen in school, we eat there with our friends. Lot of items are available there like chips, Kurkure, cold drinks.,*” (P28, F, 14 years)
Theme 9: Neighborhood food environment	“*There is a shop beside our school. So, we buy things like Kurkure from there to eat.*” (P2, M, 14 years) “*While coming back from school, I eat ice cream, chocolates. All types of snacks are available in that shop.*” (P26, M, 15 years) “*There is a cafe near my school. I buy chips, burgers, and cold drinks from there.*” (P3, F, 18 years) “*There is a general store beside my house. I secretly bring some snacks from the general store when my mother is not at home.*” (P37, M, 12 years)
Theme 10: Food and beverage marketing	“*If I see any snack advertisement on TV, I feel like eating it*……*.*” (P62, F, 17 years) “…*Food bloggers tell us about new places to eat and highlight the specialties of those restaurants. So, that also influences us to eat junk food*…*.*” (P5, F, 16 years) “*I went to the grocery store with my mother, and I saw Wai Wai Noodles there. The packaging of Wai Wai Noodles impressed me so much that I bought it from there. I had never tried it, and I thought the color of the packaging was nice, so I bought it.*” (P22, F, 15 years)

#### Theme 1: Perceptions of a snack

Varied perceptions of a snack were captured during the 62 interviews. The majority of the sample defined a snack as a ready-to-eat packaged food consumed between two meals. Another viewpoint was that snacks were mostly unhealthy foods which required no or minimal cooking and could be easily procured from street vendors or grocery stores. Our participants often consumed EDNP snacks for different reasons including satiety, for pleasure, or to relieve stress.

“*According to me, snacks are what I eat when I feel a little hungry between afternoon and evening.*” (P17, M, 13 years)

“*Snacks are like ready to eat food, which my mother doesn’t have to cook, something she can serve me instantaneously. Snacks are also supposed to be tasty that will make me feel good and not something like roti (Indian flat bread).*” (P38, F, 17 years)

#### Theme 2: Liking for unhealthy snacks

Nearly all the interviewees expressed strong liking for EDNP snacks and beverages. They savored EDNP snacks and beverages because of their taste and flavor. At the same time, participants showed aversion toward nutritious snacks like fruits and dry fruits because they did not enjoy their taste. Some of the adolescents commented that healthy foods are usually less palatable when compared to EDNP foods. Nonetheless, only a few participants reported that they liked consuming healthy snacks.

“*My favorite is Bourbon biscuit and Maggi (Brand name for instant noodles) too. I like Bourbon’s chocolatey flavor a lot. Its sweet taste is very good….*” (P19, F, 18 years)

“*My favorite snack is dry fruits. Basically, it includes almonds, cashews, figs and dates.*” (P39, M, 16 years)

#### Theme 3: Consequences of snacking

Most participants believed that snacking could have both positive and negative impacts on their health. They noted that if unhealthy snacks are consumed on a regular basis for a long duration, then it could result in various health complications including obesity and liver damage since unhealthy snacks have a high fat content and contain preservatives. In contrast, frequent intake of healthy snacks will facilitate growth and development. The participants also mentioned that snacks prepared at home were safe to consume, whereas snacks available in the market place were detrimental to health.

“*Snacking can be good as well as bad for our health. If we eat healthy food, it will be good for our health. If we eat unhealthy food, it will have bad impact on our health. But I don’t think much before eating, I just eat whatever I feel like eating.*” (P52, M, 15 years)

“*Like dry fruits are good because they do not contain chemicals.*” (P50, F, 15 years)

#### Theme 4: Snacking timing

The study participants reported that they mostly indulged in unhealthy snacking while coming back from school, traveling back from tuition classes during the evening, studying late at night, and while watching movies and television. Evening hours were recognized as the most popular period for consuming both healthy and unhealthy snacks.

“*I have some snacks in the evening.*” (P44, M, 15 years)

“*When I study late at night, then at that time I feel hungry, so instantly I eat salty chiffons or my favorite bourbon biscuits.*” (P19, F, 18 years)

#### Theme 5: Cost of snacks

During the interviews, cost emerged as an important determinant of snacking. The adolescents mentioned that ENDP snacks were available at low prices and therefore adolescents could frequently purchase and consume them as they received limited pocket money from their parents.

“*First of all, it is not expensive. It is cheap. I get less money from home, and it is tasty.*” (P10, M, 13 years)

#### Theme 6: Parental rules around snacking

All the study participants highlighted the influence of parental rules on their snacking behavior. The participants’ parents exercised strict control over their adolescents’ snacking behavior by setting food rules regarding snacking. Most parents provided healthy snacks for consumption and reprimanded their adolescents on consuming EDNP snacks and drinks. Few parents permitted snacking on EDNP food either over the weekend or once in fortnight/month. This stringent parental control often resulted in resentment among the participants, and they would subsequently break the food rules by secretly consuming snacks.

“*My parents have set a limit. But I get very angry. I feel like eating a whole packet of biscuits and chips in a day. They tell me to eat only 2 biscuits and 2-3 chocolates a week*…” (P52, M, 15 years)

“*Yeah, they (Parents) scold me a lot for eating biscuits. They say that it will have a bad impact on my health and all. But as I am a sweet lover, I go on eating those things as they are easily available and attractive.*” (P32, F, 19 years)

#### Theme 7: Influence of peers

Almost all the interviewees claimed that peers exerted a negative influence on snacking behaviors. They mentioned that their friends frequently indulged in unhealthy snacking and often incited the interviewees to consume unhealthy snacks in their company. This is reflected in the following excerpt:

“……. *they sometimes force me to eat chips, cake, burgers etc. So, I eat. They say to eat a little, but after eating a little I end up eating a lot!*” (P29, M, 19 years)

In contrast, three interviewees shared a different viewpoint about their peers. They noted that their friends consumed healthy snacks, and this motivated the interviewees to indulge in healthy snacking.

“*There are a few girls in my friend circle who are dieting, and they only eat dry fruits. They don’t eat oily snacks or junk food; they avoid all of that. They focus on maintaining a healthy diet. So, I also feel like eating healthy stuff.*” (P34, F, 17 years)

#### Theme 8: School food environment

The interviewees regarded the school as a key determinant of their snacking behaviors. First, the majority of them noted that their school canteens predominantly supplied EDNP snacks and carbonated beverages which tempted them and their classmates to purchase and consume them. Second, healthy snacks were either not available or rarely supplied in the school canteens. Third, some students reported the absence of canteens in their schools.

“*School authorities know that kids like spicy food So, they don’t offer roti and all for lunch. They know that students bring parathas [SIC: shallow fried Indian flat bread] or roti [SIC: Indian flat bread] from home for lunch. That’s why they offer different types of namkeen like Kurkure, chips etc. And spring rolls are also available in the canteen. So, the canteen has a major role, students get so many options from the canteen easily, so they choose canteen food over home-made roti and parathas.*” (P29, M, 19 years)

#### Theme 9: Neighborhood food environment

The influence of the neighborhood food environment on adolescents’ snacking behavior was often highlighted during the interviews. The interviewees mentioned that they could easily purchase EDNP snacks from convenience stores, supermarkets, street food vendors, and fast food eating venues located either in their residential or school neighborhoods as described below:

“*I always buy snacks from the supermarket Reliance Smart Bazaar. And I also buy from the general provision store next to my house.*” (P9, F, 18 years)

The study participants also claimed that they also purchased snacks from online delivery food services including Zomato, Swiggy, BigBasket, Blinkit.

“*Sometimes, I order from Zomato, especially burgers or chicken wings at midnight.*” (P31, M, 15 years)

#### Theme 10: Food and beverage marketing

The participants also discussed the influence of pervasive food and beverage marketing on their snacking behavior. They reported that food and drinks advertising on television and social media channels often influenced them to consume EDNP snacks. Some participants mentioned that they frequently received notifications on food delivery apps regarding discounts on EDNP snacks and sugary drinks. The role of social media influencers in enticing adolescents to consume snacks was also highlighted during the interviews.

“*Sometimes we get notifications on our phones. We get offers on food on Zomato and then you feel like ordering it.*” (P25, F, 19 years)

In addition, two adolescents mentioned the impact of packaging on their snacking behaviors. Attractive packaging of snacks influenced them to purchase and consume snacks as described below:

“*I once ate some crispy snack, I don’t remember the name, but its taste was very bad. I mean it smelled like rancid oil. I purchased it because its packaging was very good. I don’t remember the details, but it was probably some cartoon creature.*” (P21, M, 11 years).

## Discussion

This qualitative inquiry contributes to the limited evidence on snacking behaviors in Indian adolescents. Key findings were that adolescents primarily perceived snacks as non-nutritious, ready-to-eat food items consumed between meals. The snacking rules set by parents emerged as a facilitator of healthy snacking. The perceived barriers to healthy snacking included peers, school food services, neighborhood food environment and food and beverage marketing. These findings can be applied to inform effective and sustainable nutrition interventions specially harnessing the facilitators and overcoming identified barriers to healthy snacking and thereby potentially reducing overweight and obesity in Indian adolescents.

Overall, the respondents viewed snacks as mostly unhealthy food items which were consumed between meals, a perspective widely published in the literature (25, 61, 62). They further mentioned that unhealthy snacks were cheap, and their frequent consumption can result in various health complications. Again, similar views have been observed in previous qualitative research investigations (25, 61, 63). For example, adolescents from the neighboring country Bangladesh reiterated that less nutritious snacks were affordable, palatable and filling when compared to healthy snacks (38). However, unlike Indian counterparts, Bangladeshi adolescents expressed concerns over foodborne illnesses arising from lack of food safety and hygiene protocol followed during snack preparation, emphasizing probable cultural nuances. The socio-cultural differences in the study populations could have resulted in this disparity as the Bangladeshi evidence was primarily restricted to high school pupils attending public schools whereas the present study captured the insights of both middle and high school students from private as well as public schools. Future interventions aimed at promoting healthy snacking should make culturally relevant nutritious snacks more available, tasty, and affordable (38, 64).

Despite being aware of the health complications associated with unhealthy snacking, the study participants showed preference for unhealthy snacking as they relished the taste of unhealthy snacks over healthy snacks. They found unhealthy snacks highly palatable whereas labeled healthy snacks as boring and bland. Parallel views about the taste of unhealthy and healthy snacks were shared by American (28, 64), Irish, (25), Chinese (63) and Welsh adolescents (65). Furthermore, empirical evidence suggests that adolescents are less concerned about their long-term health and rather give more priority to instant gratification which they usually derive from consuming unhealthy snacks (38, 66). Taking this into account, government bodies might subsidize healthy snacks as well as tax unhealthy snacks. In addition, parents and school Home Economics teachers should engage adolescents in preparing tasty and healthy snacks.

Snacking rules set by parents prompted adolescents to consume healthy snacks. This facilitator of healthy snacking was also found in a US-based study in which health-oriented food rules at home were associated with healthy snacking among adolescents (67). The adolescents in our study also noted that their parents would be upset if they consumed non-nutritious snacks, again this is consistent with international findings (67). For example, a Dutch study showed that the use of restrictive snacking rules by both mothers and fathers was significantly and negatively related to adolescents’ snack intake (68). On the other hand, lack of parental food rules has been associated with unhealthy snacking among adolescents as observed in a Slovakian study (69). Some of our participants also mentioned that they often defied household snacking rules, especially in the absence of their parents, a perspective also echoed by Ecuadorian adolescents (70). To cultivate healthy snacking habits in adolescents, a systematic review recommends that parents should consistently follow the same food rules they set for their adolescents as it would help to strengthen and reinforce the family’s eating norms and values (71). Also, there is a need for parental education programs for enhancing the nutrition knowledge as well as food parenting practices of Indian parents which can positively influence adolescents’ snacking habits (72–74).

In contrast to home food rules, peers were typically viewed as significant barriers to healthy snacking. Peers mostly indulged in unhealthy snacking, and this stimulated our study participants to consume greater amounts of non-nutritious snacks when in the company of their peers, a finding observed among Danish (30), Lithuanian (75) and Iranian (41) adolescents. Interestingly, Bruening and colleagues highlighted that friendship networks are both catalysts for healthy and unhealthy eating behaviors among adolescents (76). They found significant positive associations for cereal and dairy food consumption as well as breakfast intake between adolescents and their peer groups and best friends (76). Significant positive associations were also reported between adolescents’ and their best friends’ vegetable intake, but no associations were observed among peers for fruit consumption (76).

Our interviewees also mentioned a few instances when their friends motivated them to consume healthy snacks. This suggests an important intervention opportunity. This positive peer influence could be leveraged by marketing nutritious snacks in the milieu of interaction with friends, accentuating shared experience, communication and positive emotions while snacking together (75). Another viable alternative could be the implementation of school-based Social Norms Approach (SNA) interventions (77). The SN approach rectifies misconceptions about others’ (e.g., peers) behaviors by highlighting the gap between perceived and actual norms, thereby minimizing peer pressure to conform to unhealthy behaviors such as drinking alcohol heavily (78). Adolescents from the secondary schools in the North and Midlands of England who participated in a SNA intervention were less likely to overrate their peers’ unhealthy snacking attitudes and consumed fewer EDNP snacks post-intervention and had less positive attitudes toward unhealthy snacking compared to the control group (77).

Another potential barrier to healthy snacking reported by our participants was the school food environment. They noted that there was a limited supply of healthy foods as well as widespread availability of non-nutritious snacks in their schools. This barrier has been reported widely in the literature (42, 79–83). Our finding reflects a lack of healthy canteen policies in Indian schools (84–86). Although, the Food Safety and Standards Authority of India (FSSAI) recently published regulations (Safe Food and Balanced Diets for Children in School) for Indian school canteens to provide safe and balanced diets for pupils (79, 86). Paradoxically, the present findings indicate that these FSSAI 2020 Regulations may not have been implemented in all Indian schools. Plausible reasons behind the lack of implementation of the FSSAI regulations could include lack of comprehensive and the plurality of directives (e.g., lack of heuristic coding of foods to green, yellow, red; absence of operational definition of “right portion size” with regards to all available food and beverage items) (79). Thus, more work is needed to examine the evolution of the policy in adolescent snacking behaviors. This underscores the need for rigorous implementation, regular monitoring and surveillance of these guidelines in all schools to facilitate healthy snacking among Indian school children (79).

Alternatively, healthy snacking habits could be inculcated in Indian pupils through implementation of school-based, culturally sensitive, behaviorally focused nutrition education interventions (72, 73). For yielding successful behavioral outcomes (e.g., healthy eating), nutrition education programs should be underpinned by theoretical models and culturally tailored (i.e., use of culturally appropriate behavior change initiatives) (72, 87). One such initiative is the HEAPS (Healthy Eating and Activity Program for Schoolchildren) intervention, a behaviorally focused nutrition education program underpinned by the Health Belief Model (HBM) was developed to enhance knowledge, attitudes, and practices associated with food habits and physical activity levels in adolescents aged 10–12 years from Mumbai, India (72). HEAPS was effective in reducing the daily intake of locally available deep fried Indian snacks in the participants (72). Other positive outcomes of the HEAPS intervention included increased frequency of breakfast and vegetable consumption as well as improvement in mean scores of family dietary habits (72).

Our participants often reported the easy availability and accessibility of EDNP snacks and carbonated drinks in their residential and school neighborhoods. Comparable views were highlighted in a local study conducted in New Delhi where high school and college students mentioned that ultra-processed food and sugar sweetened beverages were easily available within educational institutes as well as in their surroundings (79). Moreover, findings from a recent systematic review suggest that adolescents’ exposure to unhealthy local retail food environments was positively associated with non-nutritious food purchases including EDNP snacks and sugary drinks and subsequently unhealthy food intakes (88). In this light, food laws and regulations that limit the availability of EDNP snacks and carbonated drinks and increase the availability of healthy foods like fruits and vegetables are necessary to improve retail food environments in India and other developing countries (89). For instance, the Indian Government could launch mobile produce markets or portable fruit and vegetable markets in urban neighborhoods to increase access to fruits and vegetables in the obesogenic local food environment (90, 91).

Besides the obesogenic local food environment, digital food and beverage marketing through social media and online food delivery applications seem to have a detrimental impact on Indian adolescents’ snacking behaviors. The study participants often mentioned that they regularly receive notifications on their mobile phones from fast food restaurants regarding discounts which often tempted them to order unhealthy snacks through food delivery applications, a view also echoed by Uruguayan adolescents (92). In line with Mexican (93) and Canadian (94) research, our study also highlighted that most frequently marketed foods on social media were ready-to-eat ultra-processed snacks. According to a systematic review (95), the proliferation of digital food and beverage marketing has a consistent negative effect on adolescent eating outcomes including increased purchasing and consumption of non-core food items. This calls for implementation of digital marketing regulations to reduce adolescents’ exposure to ultra-processed food on various digital media platforms. This is most likely to involve bans on the marketing of these products, a strategy recommended by Uruguayan adolescents (92) as well as public health experts (96–98). Indian policymakers could adopt similar digital marketing regulations to those implemented in Chile, which ban the marketing of non-nutritious food and beverages to children and adolescents across different media including the internet (99). A similar regulatory policy that might be adopted is found in the Quebec province of Canada. Under its Consumer Protection Act, food advertising to individuals under 13 years of age has been prohibited (100). Recently, the Central Consumer Protection Authority of India introduced mandatory Guidelines for Prevention of Misleading Advertisements and Endorsements for Misleading Advertisements 2022 to safeguard children from marketing of unhealthy foods and drinks across all media (101). However, this policy and other existing policies have invited lot of criticism including the narrow scope of “child-directed” advertisements and absence of food classification systems for defining EDNP foods (101). Hence, robust regulations are required to protect Indian children from the harmful impacts of EDNP food marketing with clear evidence-based food classification criteria (101). Alternatively, less draconian measures involving collaborations between public health organizations and social media influencers could be promising initiatives in promoting healthy eating among adolescents (102–104).

### Strengths and limitations

Amidst the backdrop of limited evidence on adolescent snacking behaviors in the global South, this study is one of the foremost qualitative research inquiries to explore the lived experiences of urban Indian adolescents in relation to snacking behaviors and their determinants. However, the study has several important limitations. The study sample was restricted to adolescents living in one city in India and thus may not be generalized to the entire Indian adolescent population. Therefore, future research is needed to examine the lived experiences of rural Indian adolescents in relation to snacking. Additionally, the data for this inquiry was primarily based on adolescents’ responses, which may not completely replicate real-life situations in which adolescents’ snacking behaviors take place. Therefore, to gain a comprehensive view about adolescent snacking behaviors, data should be gathered from other key stakeholders like parents, school teachers and school canteen personnel. Other possible limitations which need to be addressed in future research involve the dominance of private school attendees and older adolescents in the study sample. One reason for preponderance of the former group could be that private schools are more popular than public schools in urban India (105). The dominance of older adolescents is likely to have been due to the fact that schools were closed for younger adolescents during January 2025 in Uttar Pradesh State where the study was conducted because of cold wave. Finally, no information on family income, parental education and religion was collected during the interviews, limiting the discussion of socio-economic and religious influences on adolescent snacking behaviors. Nevertheless, the participation of both public and private school adolescents in the present inquiry helped in mitigating the impact of any potential socio-economic status bias.

## Conclusion

This qualitative research inquiry contributes to the snacking literature exploring the perceptions of a sample of Indian adolescents about their snacking behaviors and its associated factors. Snacks were commonly perceived as unhealthy ready-to-eat food items consumed between meals. This research shows that multiple determinants such as parental snacking rules, peer group influences, provision of food in schools, price of snacks, local food environment, and food and beverage marketing are likely to affect Indian adolescents’ snack consumption. These determinants could potentially inform the development and implementation of effective school food policies and culturally tailored behavioral interventions to inculcate healthy snacking behaviors among nutritionally vulnerable Indian adolescents. School food environments could be improved by subsidizing healthy snacks, provision of culturally acceptable and affordable healthy snack options in school canteens and restrictions on the sale and marketing of EDNP foods both in school and across all media. Parental education could also serve as a fruitful measure in cultivating healthy snacking habits in adolescents.

## Data Availability

The original contributions presented in this study are included in this article/Supplementary material, further inquiries can be directed to the corresponding authors.
