# Supplementary material for: Factors that influence snacking behaviors among urban Indian adolescents – a qualitative inquiry
Source: Front Nutr. 2025 Sep 15;12:1637799. doi: 10.3389/fnut.2025.1637799 (PMC12478069; doi:10.3389/fnut.2025.1637799)
Supplement: Supplementary file 1 [file Data_Sheet_1.pdf]

Interview Guide

1. Tell me about your daily diet (Opening question for rapport building)
2. How would you define a snack?
3. Think about some of your “go-to” snacks. What are some reasons you snack on them?
4. Think about some of the times that you have snacked on something you didn’t really like. What were some of the reasons that you snacked on those things?
5. What do you know about the ways snacking affects your health?
6. How does where you are (i.e., home) influence what you snack on?
7. How does where you are (i.e., school) influence what you snack on?
8. Do adults around you set rules about snacking? How do you feel about those rules?
9. Tell me about some of the places you purchase snacks.
10. How does your family influence what you snack on?
11. How do your friends influence what you snack on?
